# Survey data show the correlation between school attachment dimensions and internet addiction among secondary school students in Kosovo

**DOI:** 10.1016/j.dib.2023.109638

**Published:** 2023-10-05

**Authors:** Armen Mustafa, Ertan Basha

**Affiliations:** Department of Psychology, AAB College, Pristina, Kosovo

**Keywords:** School attachment, Internet addiction, Students in Kosovo

## Abstract

This is a dataset that identifies data regarding the correlation between dimensions of school attachment and Internet addiction. The data was gained from a research population of adolescents aged 15–19 years attending secondary schools in Kosovo. The whole sample consists of 525 students, 310 (59%) of them were female, and 215 (41%) were male, respectively 214 (40.8%) were students attending the tenth grade, 189 (36%) were in the eleventh grade and 122 (23.2%) were in the twelfth grade. Data was collected via a survey with paper-pencil questionnaires from 6 different secondary schools from 4 different cities in Kosovo. Stratified and purposive sampling techniques were used. Research analyses were conducted with SPSS, using descriptive statistics and Spearman's analysis, which aimed to examine the non-parametric relationship between dimensions of school attachment and internet addiction. The research instrument was verified to have all the necessary psychometric values considered suitable for research. Several descriptive statistical analyses were performed to further clarify the data and provide the necessary platform for further analysis.

Specifications Table

**Table:** 

Subject	Developmental and Educational Psychology
Specific subject area	School Attachment and Internet Addiction
–Type of data	Primary survey data, Tables
How the data were acquired	Field survey – data was acquired using paper-pencil questionnaires from 525 respondents, the questionnaire is provided as a supplementary file.
Data format	Raw Analyzed Filtered (descriptive and inferential statistics)
Description of data collection	The data were collected through the paper-pencil questionnaires, which were delivered to secondary school students in Kosovo using purposive and stratifying sampling techniques. The questionnaires were applied to the participants in the classroom environment and took an average of 15-20 minutes.
Data source location	Region: Europe Country: Kosovo
Data accessibility	The data is with the present article and Mendeley Repository Repository name: School attachment and internet addiction Data identification number: 10.17632/syf3v93tfy.5 Direct URL to data: https://data.mendeley.com/datasets/syf3v93tfy/5

## 1 Value of the Data

• The data will be useful for researchers who want to compare the impact of school attachment and internet addiction among secondary school students, in other world countries or contribute to further meta-analysis.
• We think that the data will provide a resource for researchers who want to examine the effects of school attachment and internet addiction.
• Analyzed data provides insight for education stakeholders to become more aware of the importance of education.
• Data can be used to examine whether there is a significant relationship between the groups of variables or constructs.

## 2 Data Description

The dataset presents the understanding of school attachment and internet addiction among secondary school students in Kosovo. These data were collected throughout the school year 2022-2023, after the period of the Covid-19 pandemic. The sample consists of 525 adolescents studying at different secondary schools in Kosovo, which is not a representative sample for the whole of Kosovo, but it is representative for the secondary schools of 4 cities at a 95% confidence level and a margin of error is about 4%. 310 (59%) of the students are female, and 215 (41%) are male. 214 (40.8%) of them are students attending the tenth grade, 189 (36%) in the eleventh grade and 122 (23.2%) attend the twelfth grade. This data can be accessed on Mendeley repository [1]. Table 1 shows the socio-demographic characteristics of the students who participated in the research. Categorized Survey: contains survey questions used to collect data; Extra Datasets: All tables used in the article; Latest version of SPSS data: original data from SPSS; SPSS raw data (xlsx file): Original data .xlsx version.

**Table 1:** Socio-demographic characteristics of the population (*n* = 525).

Gender		
	Freq. (*n*)	%
Total	525	100
**Female**	310	59.0
**Male**	215	41.0
**Grade in high school**	524	100
**Grade 10**	214	40.8
**Grade 11**	189	36.0
**Grade 12**	122	23.2

Table 2 shows the data collected from the responses to the “Internet addiction scale for adolescents”. The scale was developed by Taş [2] and adapted into the Albanian language by Basha, et al. [3]. Furthermore, the internal consistency coefficient of Cronbach Alpha was found to be 0.828. The factor variances of the 9 scale items were calculated to be between 0.300 and 0.500, and the item factor loadings ranged from 0.549 to 0.708. [4]. The scale was developed in 5-point Likert type and the answers were “never”, “rarely”, “sometimes”, “often”, “always”. The scale has a single factor and consists of nine items, indicating that the high scores to be obtained from the scale are at the level of Internet addiction.

**Table 2:** Students’ responses to the items of internet addiction (*n* = 9 items).

	Never		Rarely		Sometimes		Often		Always	
	f	%	f	%	F	%	F	%	f	%
Item										
**1**. Have you ever felt anxious when you did not use the Internet?	109	20,76	123	23,42	192	36.571	67	12.762	34	6.476
**2**. Have you ever stopped yourself from using the Internet, only to find yourself back online after a while?	104	19,810	104	19,810	148	28.190	129	24,571	40	7.619
**3**. Have you ever had problems with people around you because you spend too much time online?	206	39,238	128	24,381	120	22,857	49	9,333	22	4,190
**4**. When you are not connected to the Internet, do you happen to have your mind constantly busy with things that happen on the Internet?	179	34,095	130	24,762	121	23,048	66	12,571	29	5,524
**5**. Do you constantly spend more time online than you planned?	52	9,905	74	14,095	155	29,524	166	31,619	78	14,857
**6**. Have you postponed your basic needs (eating, drinking, sleeping, etc.) due to being online for a long time?	210	40,000	90	17,143	112	21,333	76	14,476	37	7,048
**7**. In the time you spend online, have you ever let others down?	214	40,762	119	22,667	131	24,952	46	8,762	15	2,857
**8**. Have you ever used the Internet to avoid feelings and situations that bother you in life?	90	17,143	83	15,810	136	25,905	140	26,667	76	14,476
**9**. Have you had serious conflicts with family members and other people around you because you spent too much time on the Internet?	234	44,571	106	20,190	119	22,667	42	8,000	24	4,571

Table 3 shows responses to the “School Attachment Scale”, which was developed in Albanian by Aslan & Kosir (2021), which measures school attachment [5]. The reliability of the entiry scale is α = 0.737 from the combined five item sets reflecting the five dimensions of the school attachment. The scale consists of 24 questions, divided into 5 sub-dimensions: 1) Students' feelings about being in school (5 questions), 2) Students' interest in learning (6 questions), 3) Students' attitudes towards other students (5 questions), 4) Students' attitudes towards their teachers (6 questions), and 5) Students' perceptions of the school (2 questions). In the scale of school attachment, answers were given using a Likert-type scale starting with 1 (strongly disagree), 2 (strongly disagree), 3 (strongly agree), 4 (strongly agree) and 5 (strongly agree).

**Table 3:** Students’ responses to the items of attachment of school (*n* = 24 items).

		Strongly Disagree		Disagree		Neutral		Agree		Strongly Agree	
		f	%	f	%	F	%	F	%	f	%
**Item**	**Feeling about being in school**										
1	I Like School	26	4,9	23	4,3	127	24,1	232	44,1	117	22,2
2	Most mornings I look forward to going to school	52	9,9	108	20,5	139	26,4	177	33,1	49	9,3
3	I am proud to be at this school	63	12,0	42	8,0	105	20,0	273	52,0	42	8,0
4	I don't feel safe in my school	111	21,1	202	38,4	118	22,4	68	12,9	26	4,9
5	I can be myself at school	32	6,0	59	11,2	129	24,5	230	43,8	75	14,2
	**Interest in learning**										
6	Doing well in school is important to me	13	2,4	21	4,0	73	13,9	237	45,1	181	34,4
7	I care if my homework is done correctly	24	4,5	33	6,2	100	19,0	253	48,1	115	21,9
8	Most of my classes are important	27	5,1	56	10,6	83	15,8	229	43,6	130	24,7
9	I agree that school is boring	45	8,5	137	26,0	166	31,6	115	21,9	62	11,8
10	My grades matter to me a lot	40	7,6	54	10,2	110	20,9	189	36,0	132	24,9
11	I believe it is important to work hard at school	21	4,0	36	6,8	86	16,3	228	43,2	154	29,3
	**Attitudes towards other students**										
12	My classmates often annoy me	81	15,4	178	33,9	131	24,9	80	15,2	55	10,4
13	I get along well with the other students in school	14	2,6	40	7,6	125	23,8	271	51,6	75	14,2
14	I am liked by my classmates	14	2,6	27	5,1	152	28,9	236	44,9	96	18,2
15	I rarely feel lonely at school	65	12,3	162	30,8	111	21,1	134	25,5	53	10,0
16	I have many friends at school	27	5,1	76	14,4	131	24,9	191	36,3	100	19,0
	**Attitudes towards other teachers**										
17	I don't care what my teachers think of me	61	11,6	159	30,2	135	25,7	96	18,2	74	14,0
18	I get along with most of my teachers	12	2,2	21	4,0	86	16,3	259	49,3	147	28
19	I want to be respected by my teachers	6	1,1	8	1,5	54	10,2	250	47,6	507	39,4
20	I usually like my teachers	23	4,3	27	5,1	152	28,9	243	46,2	80	15,2
21	My teachers care a lot about me	56	10,6	100	19,0	198	37,7	136	25,9	35	6,6
22	I have lots of respect for my teachers	13	2,4	16	3,0	61	11,6	258	49,1	177	33,7
	**Students' perception of school**										
23	School rules and the grading system are fair	78	14,8	92	17,5	150	28,5	150	28,5	55	10,4
24	The rules at my school are applied fairly	78	14,8	113	21,5	169	32,1	123	23,4	42	8,0

## 3 Experimental Design, Materials and Methods

The data collection and analysis to carry out this research were collected with questionnaires from students who attend public secondary schools. The special focus of the research was on the relationship between school attachment dimensions and internet addiction. In order to achieve the aims and ideal results of the study, students who participated were from different cities and schools around Kosovo, using purposive and stratifying sampling. Initially, four Kosovo cities, such as Peja, Lipjani, Klina and Shtime were selected purposefully, and based on the terms of population one of the selected cities was a large city (Peja), two were medium cities (Lipjan and Klina) and one was a small city (Shtime). The total number of students in these cities was around 8,000, distributed in 11 public schools (Peja - 4 schools, Lipjan - 3 schools, Klina - 2 schools and Shtime - 2 schools). Then, 6 schools form these cities were selected to participate in the research, based on the criterion of the largest number of students per school. On this occasion, 2 schools were selected from the city of Peja (a high school and a vocational school), 2 schools in Lipjan (a high school and a vocational school), 1 school in Kline (a high school) and 1 school in Shtime (a high school). The total number of students from the 6 schools which were selected for survey was more than 5100.

Then, the classrooms from these 6 schools were selected based on stratified sampling by taking the same number of classrooms for each grade in each school. The selection of classrooms was done randomly, taking the classroom 1 and 5 for each grade. However, the response rate was not the same for each grade, about 80% for the tenth grade, 70% for the 11th grade and 49% for the 12th grade, totally the rate of responses were 66%. The students from the classes that have been selected to participate in the research have been informed in advance that the survey will be conducted on the specified day, informing them about procedures of the survey and that their participation is anonymous, confidential, voluntary and not mandatory, which means that at any stage they could leave the process.

Those surveys were collected in the school year 2022-2023. The questionnaires were administered to different age groups starting from the age of 15. On the day of administration of the questionnaires, the students were explained again about the importance of the study and asked to answer the items sincerely in the questionnaire. They were also told that the study is anonymous, and confidential, so they did not have to provide credentials such as their names or any other identifying information. In the end, data were collected from 525 participants in total. 4 of the data were not subjected to the analysis because they were incomplete. Research analyzes were conducted with IBM SPSS Statistics and Spearman's analysis, which aimed to examine a non-parametric relationship between school attachment and internet addiction (Table 4). Relationship screening models are studies that aim to determine the change between two or more variables [6].

**Table 4:** Correlations between the groups of variables.

		Correlations					
		Internet addiction	Feel about School	Interest in Learning	Attitude to Students	Attitude to Teachers	Students’ Perception of School
Internet addiction	Pearson Correlation	1	-,283**	-,138**	,058	-,123**	-,274**
	Sig. (2-tailed)	,	,000	,002	,188	,005	,000
	N	525	525	525	525	525	525
Feeling about School	Pearson Correlation	-,282	1	,321**	,084	,333**	,400**
	Sig. (2-tailed)	.000	.	.000	.000	.000	.000
	N	525	525	525	525	525	525
Interest in Learning	Pearson Correlation	-,138**	,321**	1	,247**	,436**	,321**
	Sig. (2-tailed)	,002	,000	.	.000	.000	.000
	N	525	525	525	525	525	525
Attitude to Students	Pearson Correlation	,058	,084	,247**	1	,271**	,049
	Sig. (2-tailed)	,188	,054	.000	.	.000	.261
	N	525	525	525	525	525	525
Attitude to Teachers	Pearson Correlation	-,123**	,333**	,436**	,271**	1	,363**
	Sig. (2-tailed)	,005	,000	,000	,000	.	.000
	N	525	525	525	525	525	
Students’ Perception of School	Pearson Correlation	-,274**	,400**	,321**	,049	,363**	1
	Sig. (2-tailed)	.000	.000	.000	,261	.000	.
	N	525	525	525	525	525	

## Ethics Statements

Ethical approval was obtained from the Code of ethics for scientific research of the AAB College in Prishtina, Kosovo with the protocol number of 271/23. Respondents’ participation was completely consensual, anonymous, and voluntary. The collecting data was conducted according to the Declaration of Helsinki.

In addition to the ethical approval from the Code of ethics, consent was also obtained from the schools that participated in the study and Parent's Councils at the school level, who were informed in detail about the objectives of the study, and that the study will be completely voluntary, anonymous and confidential. With the consent of the schools, it was ensured by them also support of the school psychologist during the time of administration of the questionnaires.

Then, to include students' in decision making for participating in the study and to get their consent, schools have also informed them that the study will be carried out on the specified day, informing them about procedures of the study and about that their participation is anonymous, confidential, voluntary and not mandatory.

Students who were present in the classroom in the time of administration of the questionnaires, before the administration of questionnaires, were again explained about the procedures and that their participation is voluntary, anonymous, and confidential, and that they can leave the process before starting the administration of questionnaires or at any stage of the process. The students who stood at classrooms meant they consent the participation in survey.

## Limitations

As it was mentioned above, the data were collected in the school year 2022–2023, after the period of the Covid-19 pandemic. This can be considered as a limitation because according to the data obtained, the pandemic had a negative effect on school attachment and internet addiction. Among the reasons for this, the remote working, distance education and online socialization during the quarantine period caused an increase in internet addiction as a communication and connection tool and weakening of connections with the school. During the pandemic, people relied heavily on the internet to access information, entertainment and essential services, and there may be an increase in internet addiction. It is important to note that the effects of the pandemic on school attachment and internet use are complex and may continue to evolve as societies adapt to new circumstances.

Another limitation of these data is that the selected sample is not representative for all secondary schools in Kosovo and this does not allow the generalization for all secondary schools in Kosovo. However, the sample is representative for the respective cities and schools, which were selected for the administration of the questionnaires.

Another one of the limitations of the data may be the fact that the participants were 59% female students and 41% male students, since from a demographic point of view students aged 15–18 years in schools in Kosovo have more male students than females. However, the schools that have been selected to be part of the study in most cases have been high schools (not vocational schools), where approximately 57% of students in high schools in Kosovo are female.

When this is added to the fact that the participation in the study was voluntary, with an overall response rate of about 66%, which resulted in this gender percentage of the participants and with a lower responses rate of 12th grade students. This can be considered as another limitation.

## CRediT authorship contribution statement

**Armen Mustafa:** Investigation, Validation, Resources, Supervision, Writing – review & editing. **Ertan Basha:** Methodology, Visualization, Conceptualization, Writing – original draft.

## Data Availability

School attachment and internet addiction (Original data) (Mendeley Data).
